# The PI3K/Akt Pathway in Tumors of Endocrine Tissues

**DOI:** 10.3389/fendo.2015.00188

**Published:** 2016-01-11

**Authors:** Helen Louise Robbins, Angela Hague

**Affiliations:** ^1^Department of General Surgery, University Hospital Coventry and Warwickshire, Coventry, UK; ^2^School of Oral and Dental Sciences, School of Cellular and Molecular Medicine, University of Bristol, Bristol, UK

**Keywords:** thyroid tumors, parathyroid tumors, pituitary tumors, adrenocortical carcinoma, phaeochromocytoma, neuroblastoma, gastroenteropancreatic neuroendocrine tumors, Akt/PKB kinases

## Abstract

The phosphatidylinositol 3-kinase (PI3K)/Akt pathway is a key driver in carcinogenesis. Defects in this pathway in human cancer syndromes such as Cowden’s disease and Multiple Endocrine Neoplasia result in tumors of endocrine tissues, highlighting its importance in these cancer types. This review explores the growing evidence from multiple animal and *in vitro* models and from analysis of human tumors for the involvement of this pathway in the following: thyroid carcinoma subtypes, parathyroid carcinoma, pituitary tumors, adrenocortical carcinoma, phaeochromocytoma, neuroblastoma, and gastroenteropancreatic neuroendocrine tumors. While data are not always consistent, immunohistochemistry performed on human tumor tissue has been used alongside other techniques to demonstrate Akt overactivation. We review active Akt as a potential prognostic marker and the PI3K pathway as a therapeutic target in endocrine neoplasia.

## Introduction

The phosphatidylinositol 3-kinase (PI3K)/Akt pathway is recognized as a key pathway in carcinogenesis, with activating mutations in the *PIK3CA* gene (encoding the p110 catalytic subunit of PI3K) or inactivating mutation in the *PTEN* gene (encoding phosphatase and tensin homolog deleted on chromosome ten) occurring commonly in diverse human tumors (1). The PI3K/Akt pathway is explained in depth below; however, in brief: the actions of PI3K lead to phosphorylation (and hence activation) of Akt to p-Akt, an effect that is antagonized by PTEN. Akt represents a key signaling node: it phosphorylates a plethora of downstream cytoplasmic and nuclear targets, connecting it to a multitude of interrelated signaling pathways, and therefore it is responsible for modulating multiple processes – including cell survival, cell cycle progression, DNA repair, protein synthesis, glucose metabolism, differentiation, angiogenesis, and cellular migration (2–5). The central role of PI3K/Akt signaling in this complex network of cellular processes makes this pathway of great importance in cancer cells, and indeed p-Akt is known to be overexpressed in a multitude of human cancers, and overexpression appears to be related to poor overall survival in some cancer types (6). The PI3K/Akt pathway is perhaps less well studied in tumors of endocrine tissues than in other, more common, malignancies. Nonetheless, there is growing evidence from both human tumors and animal models that this pathway may play a significant role in tumors of endocrine tissues. In this review, we seek to explore the evidence relating to the role of the PI3K/Akt pathway in tumors of endocrine tissues, with particular focus on evidence from immunohistochemical studies.

## PI3K/Akt Signaling Pathway

**Figure 1:**
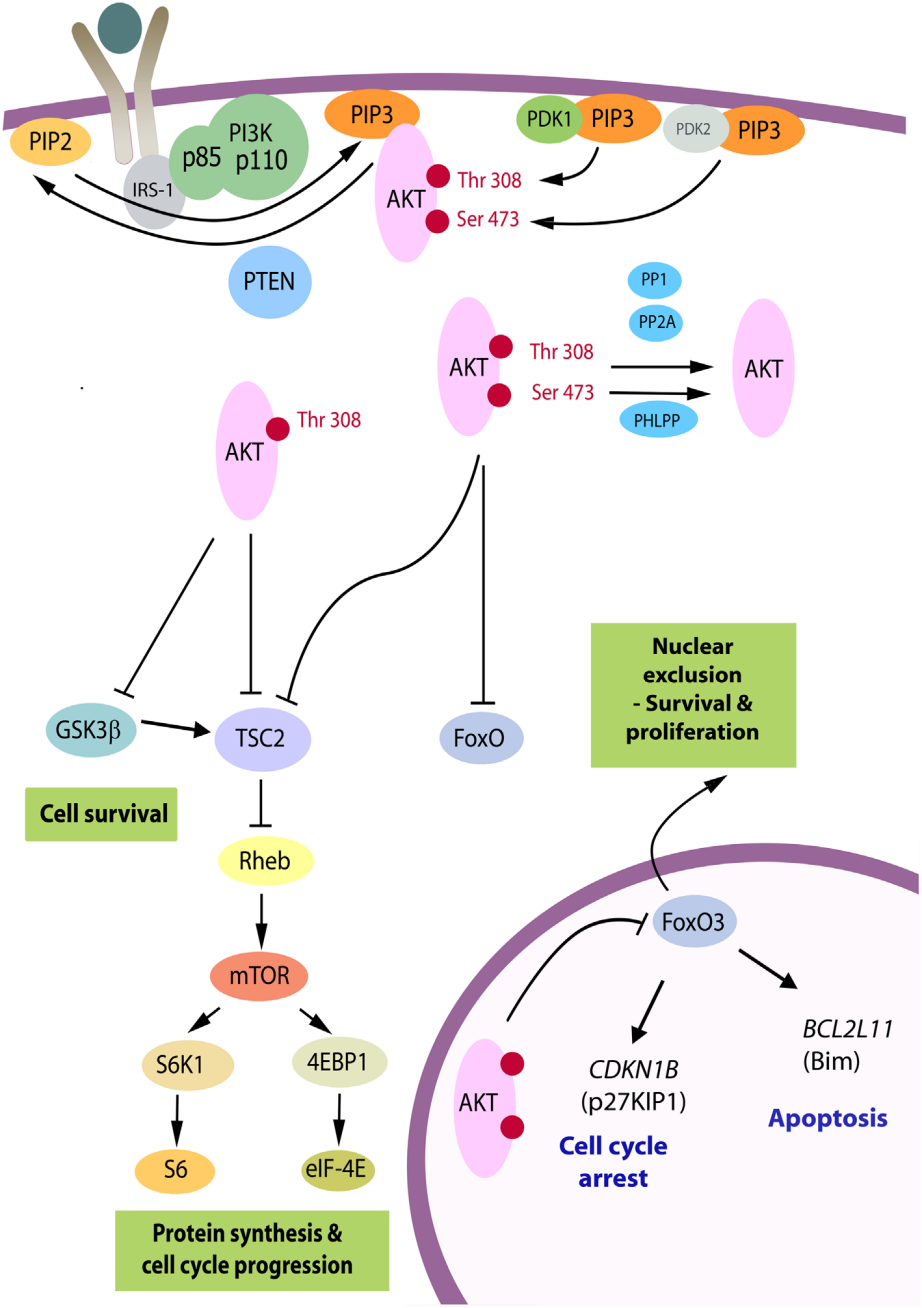
**The PI3K/Akt pathway**. Akt is recruited to the plasma membrane by phosphatidylinositol (3,4,5) trisphosphate (PIP3) produced from phosphatidylinositol (4, 5) bisphosphate (PIP2) by phosphatidylinositol 3-kinase (PI3K). PTEN is a phosphatase that promotes the reverse reaction. For activation, Akt is phosphorylated on Thr(308) by phosphoinositide-dependent kinase 1 (PDK1) and on Ser(473) primarily by the mTORC2 complex serving as PDK2. Upon full activation, Akt leaves the membrane and can adopt a nuclear or cytosolic localization. There are over 70 known molecular targets of the Akt kinase and the three targets believed to be the most important in carcinogenesis are shown. By phosphorylation of TSC2, Akt relieves its repressive effects on Rheb, resulting in downstream activation of the mTORC1 complex and enhanced RNA translation. GSK3β is a tumor suppressor, which targets a number of proliferation and survival regulators, including β-catenin and Mcl-1, and elevates activity of TSC2 [reviewed in Ref. (9)]. The family of forkhead transcription factors, FoxO, upregulates genes controlling cell cycle arrest and apoptosis and is inhibited by Akt. Whereas GSK3β and TSC2 can be phosphorylated by p-Akt(Thr308) in cells, phosphorylation at Ser(473) is critical to inactivate FoxO proteins [reviewed in Ref. (8)]. When these are phosphorylated they become transcriptionally inactive and subject to nuclear export. FoxO3A exhibits reduced nuclear expression in the majority of thyroid cancers in association with high levels of p-Akt(Ser473), and relevant FoxO3A downstream target genes have been identified in follicular rat thyroid cells (10) and subsequently in benign (FRTL-5) and malignant human thyrocytes (FTC-133) (11), as the *CDKN1B* gene encoding cyclin-dependent kinase inhibitor, p27^KIP1^, and *BCL2L11*, encoding Bim, a pro-apoptotic member of the Bcl-2 family of proteins.

PI3K/Akt signaling in human cancer can be driven by tyrosine kinase receptors, G-coupled protein receptors, or mutant *RAS*. PI3K catalyzes the production of the lipid second messenger, phosphatidylinositol (3,4,5) trisphosphate (PIP3) from phosphatidylinositol (4,5) bisphosphate (PIP2). The action of PI3K is antagonized by PTEN, which dephosphorylates PIP3 at the 3′ position, returning it to its inactive form. Akt specifically binds the 3′-phosphorylated inositol lipids via its plekstrin homology domain, hence PIP3 recruits Akt to the cell membrane. Akt–PIP3 binding results in a conformational change that opens up the C-terminal kinase domain of Akt for activation by phosphorylation. The Akt family comprises three highly homologous serine-threonine kinases: Akt1, Akt2, and Akt3. For activation, each isoform requires phosphorylation at equivalent threonine and serine residues in the Akt1/2/3 molecule: first, close to the active site at Thr308/309/305 in a region termed the activation loop mediated by phosphoinositide-dependent kinase 1 (PDK1); and second, in a C-terminal hydrophobic motif at Ser473/474/472 (Figure 1). The kinase responsible for the serine phosphorylation has been debated, and kinases that can phosphorylate at this site include integrin-linked kinase (ILK), protein kinase Cβ2, DNA-dependent protein kinase (DNA-PK), and ataxia telangiectasia mutated (ATM) (7). Akt can also autophosphorylate at this residue if the plekstrin homology domain is exposed (allowing recruitment to the plasma membrane) and the threonine residue (Thr308 in Akt1) is phosphorylated (8). However, the mTORC2 complex is now believed to be the main kinase responsible for phosphorylation at the Ser473/474/472 position.

Akt is phosphorylated at multiple sites by PI3K-independent mechanisms, for example tyrosine kinases such as Ack1/TNK2, Src, and protein tyrosine kinase 6 (PTK6) and serine/threonine kinases such as IKKϵ, TANK-binding kinase 1 (TBK1), Mre1/ATM, or DNA-PKcs. These modifications may alter Akt conformation and contribute to cancer development or tumor resistance to inhibition of PI3K [reviewed in Ref. (12)]. Recently, cyclin A/cyclin-dependent kinase-2 complexes have been reported to phosphorylate Akt1 at S477 and T479, stabilizing the C-terminal tail and promoting its kinase activity and ability to protect against apoptosis. Hence, Akt activity may be intimately linked with the cell cycle. S477/T479 phosphorylation may also prime Akt1 for mTORC2-mediated phosphorylation at S473 (13).

Akt is negatively regulated by the Pleckstrin homology domain leucine-rich repeat protein phosphatases 1 and 2 (PHLPP1/2), which selectively dephosphorylate Akt at the Akt1 Ser(473) site, while protein phosphatase PP2A dephosphorylates Akt at the Thr(308) site (8). PHLPP1 and PHLPP2 show relative specificity for different Akt isoforms. For example, in pancreatic adenocarcinoma cells, PHLPP1 selectively dephosphorylated Akt2, whereas PHLPP2 selectively dephosphorylated Akt1 (14).

In addition to its activation at the plasma membrane, Akt can be activated by nuclear pools of PI3K, and PDK1 and DNA-PK (15). In this regard, it is unsurprising that Akt is considered a signaling hub for tumor cells and the PI3K/Akt/mTOR pathway in particular has been the interest of targeted drug development (16).

Mammalian target of rapamycin (mTOR) is a serine/threonine kinase that forms two complexes, mTOR complex 1 (mTORC1) and mTOR complex 2 (mTORC2), implicated in carcinogenesis. Akt removes inhibition of mTORC1 (consisting of mTOR, mLST8, Raptor, PRAS40) by targeting the negative regulators tuberous sclerosis protein 2 (TSC2) and proline-rich Akt substrate of 40 kDa (PRAS40) by direct phosphorylation (4) and by indirect inhibition of TSC2 through GSK3β phosphorylation [reviewed in Ref. (17)]. mTORC1 phosphorylates ribosomal S6 kinase (70 kDa S6K1 or p70RSK), and eIF4E-binding proteins (4E-BPs) to enhance protein synthesis. mTORC2 (consisting of mTOR, mLST8, Rictor, Sin1) can act as the PDK2 for Akt and phosphorylate it at serine 473, but is not thought to function downstream of Akt (17).

There are a number of negative feedback mechanisms that control signaling through the PI3K/Akt/mTOR pathway that need to be considered when designing drug targeting strategies for human cancer. mTOR inhibition induces activation of Akt signaling pathways, through elimination of negative feedback loops. Ultimately, this may lead to growth escape and drug resistance, limiting the effectiveness of mTOR inhibition, and therefore combination treatments could be promising (18–20). The feedback loops that normally quench Akt signaling have been reviewed by Rozengurt et al. (21). mTOR inhibition prevents negative feedback mechanisms; in one of these S6K1 phosphorylates IRS-1 (insulin receptor substrate-1) causing it to dissociate from tyrosine kinase receptors; in another mTORC1 phosphorylates GRB10 which suppresses insulin and IGF receptor kinase activity and facilitates their degradation; and in a third mTORC2 assembly is prevented by phosphorylation of Sin1, mediated by S6K or Akt depending on the cell type. Whereas mTORC1 is sensitive to inhibition by rapamycin (sirolomus), mTORC2 is generally insensitive meaning Akt phosphorylation at Ser473 is enhanced with treatment because of inhibition of the latter feedback loop, although in some cell types long-term treatment with rapamycin can block mTORC2 assembly [reviewed in Ref. (22)]. Active site mTOR inhibitors function against mTORC1 and mTORC2, reducing phosphorylation of Akt at Ser473 but enhancing that at Thr308. Dual active site inhibitors of PI3K and mTOR are therefore useful to damp down the activation of the pathway; however, these elicit compensatory signaling through the MEK/ERK pathway. Hence researchers are currently investigating the potential for combining inhibition of tyrosine kinase receptors with mTOR targeting (21).

## Immunohistochemical Analysis of Akt Activation

Immunohistochemistry for p-Akt has been used to assess the relevance and implications of the activation of the PI3K/Akt pathway in human cancers. Activation of Akt may be assessed through use of phospho-specific antibodies, raised against either phospho-Ser473-Akt or phospho-Thr308-Akt. These antibodies recognize all three phosphorylated Akt isoforms (23). Caution needs to be taken in assessing phospho-specific protein detection by immunohistochemistry, since delayed fixation can reduce the efficiency of staining and larger tumors can sometimes appear negative in the center of the fixed sample (24, 25). With this in mind, p-Akt has been used to determine a snapshot of Akt signaling in tumor specimens, with a view to its utility as a prognostic or predictive marker.

Ocana et al. (6) conducted a meta-analysis of reports in the literature of PI3K/Akt activation in solid tumors and overall survival. Eligibility criteria were the availability of survival data for at least 5 years in relation to three types of pathway aberrations (i) mutations in the *PI3K* gene, (ii) lack of PTEN expression by immunohistochemistry or western-blot, or (iii) evaluation of downstream components of the PI3K/Akt pathway such as Akt itself or downstream targets including phospho-S6, mTOR, phospho-mTOR, or phospho-4EBP1 by immunohistochemistry. Overall, they found an association between defects in the PI3K/Akt/mTOR pathway and poor 5-year survival. While the number of tumor types for which this information was available was small, the association was more marked in gastrointestinal tumors and gynecologic cancers than others. A recent meta-analysis of breast cancer patients showed significant association between p-Akt overexpression and worse overall survival (6,349 patients from 20 studies) and worse disease-free survival (8,683 patients from 24 studies) (26). There was no significant difference between patient groups according to stage of cancer, estrogen receptor status, progesterone receptor status, and HER2 status. Between-study heterogeneity was attributed by the authors at least in part to different scoring methods for p-Akt status and definitions of p-Akt overexpression. They called for a standardized assay methodology, which would help to determine whether Akt inhibition is likely to be an effective targeted cancer treatment.

The number and size of studies investigating the activation of the PI3K/Akt pathway in cancers of endocrine tissues are small and do not include 5-year overall survival data, but consideration of what is known on this subject will help to define the relevance of defects in this pathway to carcinogenesis and future approaches to therapy.

## Thyroid Cancer

As the most common cancer of endocrine tissues, thyroid carcinomas are the most well studied. Immunohistochemical studies are corroborated by genetic analysis and animal models to provide strong evidence for an important function of the PI3K/Akt pathway in thyroid carcinogenesis. Thyroid carcinoma includes well-differentiated thyroid cancers of the papillary, follicular, and Hurthle cell types (of which papillary tumors are the most common), and undifferentiated or anaplastic thyroid cancer. Although uncommon, anaplastic thyroid cancer is aggressive with a high mortality rate and is believed to arise by anaplastic transformation from pre-existing follicular and papillary tumors through distinct genetic pathways (27). These cancers are derived from the follicular cells, whereas medullary carcinoma is derived from the C cells of the thyroid. A role for the PI3K/Akt pathway in thyroid carcinogenesis was first suggested by propensity of patients with Cowden’s syndrome, an autosomal dominant multi-organ hamartoma syndrome, to develop thyroid tumors. Germline mutations in *PTEN* underlie 80% of Cowden’s syndrome cases. Other cases result from germline promoter hypermethylation resulting in transcriptional down-regulation of *KILLIN*, a pro-apoptotic gene sharing *PTEN* promoter sequences, or germline mutations and variants in the succinate dehydrogenase genes (*SDHx*), which result in elevated Akt and MAPK signaling (28), or mutation in either *PIK3CA* or *AKT1* (29).

**Figure 2:**
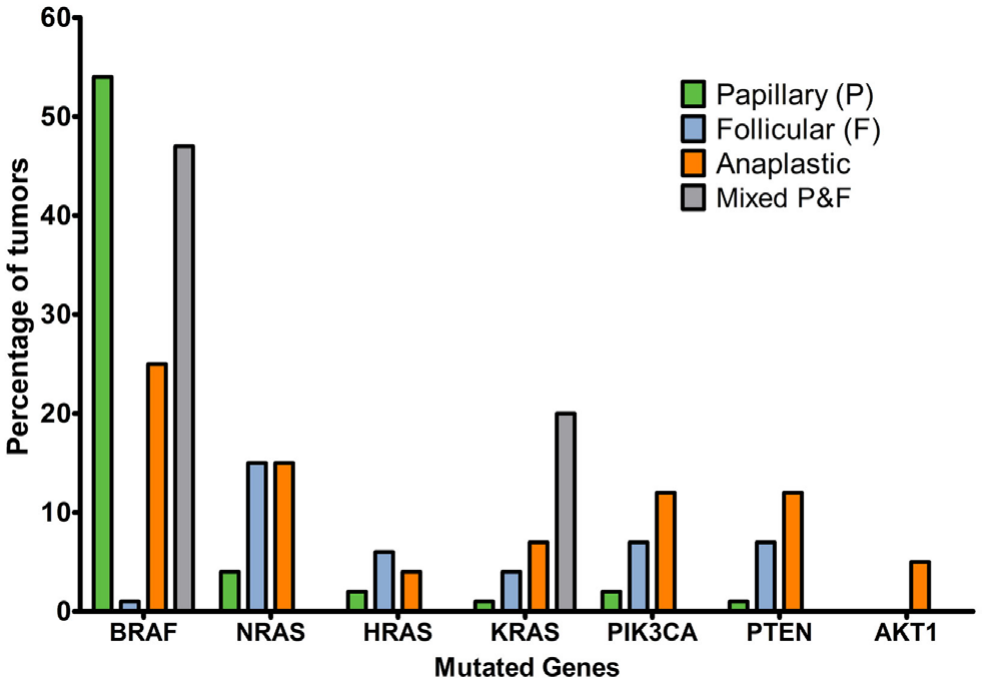
**Percentage of tumors with mutations affecting MAPK or PI3K/Akt signaling pathways in thyroid cancer histotypes of follicular cell origin**. Data obtained from the COSMIC, 2014 (32) database considering the top 20 mutations (accessed 16/12/2015). Numbers of samples: papillary, 19239; follicular, 253; anaplastic, 564; mixed papillary and follicular, 69.

The Cancer Genome Atlas Network reported that 61.7% of papillary thyroid cancer tumors had *BRAF* mutations, with a predominance of V600E substitutions (30) and 12.9% had mutations in *RAS* genes. “*BRAF*V600E-like tumors” had gene signatures distinctly different to “*RAS*-like tumors.” The former signal through MAPK to strongly activate ERK, whereas the latter signal through MAPK preferentially via c-RAF and additionally activate the PI3K pathway. The Catalogue of Somatic Mutations in Cancer (COSMIC, 2014, 31, 32) indicates that the incidence of *BRAF* mutation is strikingly higher in papillary than follicular or anaplastic cancers and that mutations in the *RAS* genes are more common in papillary thyroid cancers. Mutations in the PI3K/Akt pathway are a feature of follicular and anaplastic tumors and are less frequent in papillary tumors (Figure 2). Ricarte-Filho et al. (33) found *BRAF* mutation to be more common in radioactive iodine-resistant recurrent or metastatic thyroid carcinomas compared with poorly differentiated primary tumors, which more frequently harbored *NRAS* mutations. They reported that *AKT1* mutations were detected only in metastases and that different metastases from the same individual could exhibit *PIK3CA* mutations or *AKT1* mutations, leading them to suggest that defects in the PI3K/Akt pathway occur during tumor progression, and that use of primary cancer material to stratify patients for specific therapies may not be the most effective strategy. However, mutation tells only part of the story, and changes in gene copy number, expression and protein stability may contribute to over-activation of the PI3K/Akt pathway.

## Animal Models of Thyroid Cancer

Rodent models have provided evidence for elevated PI3K/Akt signaling in thyroid carcinogenesis. *PTEN*^+/−^ mice serve as a model of Cowden’s disease and develop diverse types of tumor including prostate, endometrium, thyroid, adrenal medulla, and small intestine tumors. Chen et al. (34) crossed *PTEN*^+/−^ mice with *Akt1^−/−^* mice to determine whether this would attenuate the propensity for tumor development. While the most marked reduction in tumor incidence was observed in the prostate, endometrium, and small intestine, Akt deficiency significantly reduced the incidence of thyroid tumors and high grade tumors of the adrenal medulla. Mice in which thyroid follicular cell specific *PTEN* deletion is achieved by thyroid peroxidase *Cre*-mediated recombination exhibit a marked increase in follicular cell p-Akt(Ser473) immunoreactivity and proliferation and develop follicular adenomas (35). The Akt target FoxO1, the mTOR target p70S6K1, and downstream S6 were highly phosphorylated in the mutant thyroids. Although *PTEN*^+/−^*PHLPP1^−/−^* mice are available (36), the role of PHLPP in thyroid and adrenal medulla carcinogenesis has yet to be explored. Neither have any studies addressed PHLPP expression in human thyroid cancer.

The TRPV mouse model makes use of the fact that the thyroid hormone receptor (TR) is a tumor suppressor for thyroid cancer (37). The PV mutation results in loss of thyroid hormone binding to the receptor and transcriptional activity and a knock-in mouse carrying two mutant TR alleles (*TR*β*^*PV/PV*^*) exhibited spontaneous follicular thyroid carcinomas, while *TR*β*^*PV/*^*^+^ heterozygous mice did not. Kim et al. (38) examined subcellular localization of Akt in the thyroid of such mice by both immunohistochemistry and confocal microscopy. All three Akt isoforms were overexpressed in the thyroid cancers of older *TR*β*^*PV/PV*^* mice compared to age-matched wild-type controls. In primary tumors, Akt1 was chiefly in the nucleus, Akt2 and Akt3 were primarily cytosolic and p-Akt(Ser473) was both nuclear and cytoplasmic. In metastases, all three Akt isoforms and p-Akt(Ser473) were primarily nuclear. Functional studies of the PI3K/Akt pathway were carried out by treating primary *TR*β*^*PV/PV*^* thyroid cells with the PI3K inhibitor LY294002 or transducing them with dominant negative Akt, both of which reduced cell motility in transwell assays.

Pringle et al. (39) generated mice with thyroid-specific knockout of both *PRKAR1A* and *PTEN* genes. *PRKAR1A* encodes the type 1A regulatory subunit of protein kinase A (PKA); when this gene is inactivated, PKA activity is enhanced. Single *PRKAR1A* knockout (*R1a-TpoKO*) mice develop thyroid cancer, but do not exhibit metastases. By contrast, the *PRKAR1A*-*PTEN* double knockout (*DRP-TpoKO* mice) developed aggressive follicular carcinomas, which frequently metastasized to the lungs. *PTEN* single knockout thyroids (*pten-TpoKO*) only developed benign follicular hyperplasia, but there was diffuse nuclear and cytoplasmic p-Akt staining and increases in phosphorylated mTOR and p70S6K. p-Akt staining in *DRP-TpoKO* tumors exhibited membranous localization. Pringle et al. (39) hypothesized that PKA may redirect Akt to a membranous localization and that, independently, PKA may contribute to the activation of mTOR. This is consistent with their immunohistochemical analysis suggesting that a higher proportion of follicular carcinomas overexpress p-p70S6K than p-Akt, although it would be useful to expand the number of follicular carcinomas analyzed.

While most studies of p-Akt have utilized genetic mouse models, a rat two-stage chemically induced thyroid cancer model has also been informative. The thyroid carcinogen *N*-bis(2-hydroxypropyl)nitrosamine followed by promotion using sulfadimethoxine for 10 and 15 weeks was sufficient to induce focal follicular cell hyperplasia, follicular cell adenomas, and carcinomas (40). This study compared staining using Akt1, Akt2, p-Akt(Ser473), and p-Akt(Thr308) antibodies as well as antibodies to PTEN, p-PTEN, GSK3β, and p-GSK3β(Ser9). Staining intensity and incidence for Akt1 and Akt2 were increased in carcinomas compared to focal follicular cell hyperplasia, as was p-Akt(Ser473) and p-Akt(Thr308). PTEN and GSK3β are both inactivated by phosphorylation. Although PTEN staining was higher, so was staining for inactive p-PTEN, suggesting that inactive p-PTEN accumulation may underlie Akt activation in these tumors. The elevation of p-GSK3β(Ser9) staining intensity observed in the tumors was further indication of increased Akt activation.

## Evidence for Akt Activation in Human Thyroid Carcinoma

There is growing evidence from examination of Akt phosphorylation that Akt signaling has a role in sporadic thyroid cancers [reviewed in Ref. (40, 41)]. Ringel et al. (42) examined levels of p-Akt(Ser473) and Akt1, Akt2 and Akt3 in 8 follicular thyroid cancers, 9 papillary thyroid cancers, and 11 normal thyroid samples by western blotting and observed increased Akt1, Akt2 and p-Akt(Ser473) in follicular but not papillary cancers. By contrast, also using western blotting, Miyakawa et al. (43) compared seven matched pairs of papillary thyroid cancer and adjacent normal tissue and found total Akt levels unchanged, but there was significantly higher p-Akt in tumor tissue compared to surrounding tissues (five of seven pairs), with elevated levels of activated downstream targets p-Bad and p-p70S6K. Mandal et al. (44) also found elevated levels of p-Akt(Ser473) in six out of eight papillary thyroid cancers compared to adjacent normal tissue, whereas total Akt levels were similar. Krzeslak et al. (45) used an ELISA method, and found enhanced levels of cytoplasmic Akt1 in 23 thyroid carcinomas compared with 16 non-neoplastic lesions (nodular goiters). The majority of Akt1 was cytosolic in differentiated cancers whereas two of three anaplastic cancers showed lower cytosolic and higher nuclear Akt1 expression. However, when Akt1 immunoprecipitates were examined by immunoblotting, the ratio of p-Akt(Ser473) to total Akt1 was lower in cancers compared to normal tissues (although only a subset of the tumors were shown to be analyzed in this way and it is unclear if the anaplastic cancers with high nuclear Akt1 were included). On this basis, the authors proposed that either Akt2 or Akt3 might be activated in thyroid cancers rather than Akt1.

Levels of PTEN regulate the extent to which Akt is phosphorylated. Exogenous overexpression of PTEN in ARO or FB-1 cells caused decreased p-Akt(Ser473) and increased p27^KIP1^ expression leading to cell cycle arrest with no evidence of apoptosis. In a panel of 13 human thyroid carcinomas, there appeared to be an inverse correlation between PTEN and p-Akt(Ser437) levels as measured by western blotting, although with low numbers this was not quantified and analyzed statistically (46). Gimm et al. (47) stained 139 benign and malignant non-medullary thyroid tumors for PTEN and measured loss of heterozygosity in 92 tumors informative at the *PTEN* locus. This generated a picture of progressive loss of staining and increased frequency of *PTEN* allelic loss with progression to malignancy and an aggressive phenotype.

Results of p-Akt immunohistochemistry studies of human thyroid cancers are summarized in Table 1. Papillary, follicular, and anaplastic thyroid cancers exhibit increased p-Akt(Ser473) expression compared with normal tissue (48–50). One study reported clear p-Akt(Ser473) staining in only a minority or thyroid cancers, but this discrepant result appears to be due to cytoplasmic staining being judged as non-specific in this report (51). Detection of p-Akt(Ser473) in benign follicular adenomas is uncommon, but it is detectable in atypical cells of atypical adenomas, and frequent in follicular thyroid cancer – suggesting that Akt activation may play a role in the malignant phenotype (48, 52). This is further supported by the finding that p-Akt(Ser473) expression is greater at the invasive front of cancers, and also increased in cells invading the vasculature (48, 53).

**Table 1:** **Results of Immunohistochemical staining studies for p-Akt(Ser473) in thyroid tumors**.

Antibody	Specimens	Key results	Author (reference)
**Tumours arising from follicular cells**			
**p-Akt(Ser473)** *CST 1:50*	115 PTC on TMA (Korean cohort)	10 cases showed clear nuclear staining (cytoplasmic p-Akt staining observed in approximately half of the samples was considered to be non-specific)	Shin et al. (51)
**p-Akt(Ser473)** *CST 1:100*	66 normal thyroid 20 FA 10 FTC 26 PTC 10 follicular variant papillary (samples from Ukraine and Tokyo)	p-Akt staining rare in normal thyroid samples (4/66). Nuclear p-Akt in 8/20 FA in areas near vessels or in atypical cells and in FTC regions of capsular and vascular invasion (10/10) Both nuclear and cytoplasmic p-Akt in invasive regions and lymph node metastases of all PTC compared with adjacent normal tissue. Only 2/8 FVPC p-Akt positive	Vasko et al. (48)
**p-Akt(Ser473)**	38 ATC (samples from Spain and Italy)	p-Akt detected in high proportion of anaplastic thyroid cancers, associated with proliferation. Only focal staining in normal tissue	Garcia-Rostan et al. (49)
**p-Akt(Ser473)** *CST 1:250*	100 thyroid tumors (10 FA, 62 PTC, 23 FTC, 5 ATC) (Italy cohort)	Expression more common in carcinomas than benign tumors. Associated with cytoplasmic p27^KIP1^ localization, but not with tumor differentiation	Motti et al. (52)
**p-Akt(Ser473)**	8 PTC, 8 HT, 8 HT + PTC, 34 normal (samples from USA)	Hashimoto’s thyroditis and cancer showed increased p-Akt. p-Akt higher in cancer with Hashimoto’s thyroiditis than cancer alone	Larson et al. (50)
**p-Akt(Ser473)** *CST*	536 PTC on TMA (Saudi Arabian cohort)	Paper reports on c-Met. p-Akt staining referred to as data not shown. 55% p-Akt positive; significantly associated with c-Met overexpression	Siraj et al. (54)
**p-Akt(Ser473)** *CST*	27 ATC	16 (59.3%) p-Akt positive	Liu et al. (55)
	25 FTC (samples from USA)	16 (64%) p-Akt positive	
**p-Akt(Ser473)** *CST*	26 ATC (samples from USA)	p-Akt detected in 22/26 anaplastic thyroid carcinomas. All had cytoplasmic staining and 17 of them had nuclear. Associated differentiated thyroid carcinoma, where present, exhibited only cytoplasmic p-Akt	Santarpia et al. (56)
**p-Akt(Ser473)** *CST*	463 PTC on TMA (Saudi Arabian cohort)	255/463 overexpressed p-Akt	Uddin et al. (57)
		Fatty acid synthase expression significantly associated with p-Akt expression	
**p-Akt** *Rabbit mAb CST 1:500*	196 benign and malignant tumors (65 FA, 68 FTC, 63 PTC)	Strong p-Akt staining intensity in 65% of FTC and in 70% of PTC. Very faint and present in <13% of FA and <5% of normal thyroid. Positive correlation with cytoplasmic FoxO3A staining.	Karger et al. (10)
	10 normal (samples from Germany)		
**p-Akt(Ser473)** *CST*	2 ATC versus 23 normal thyroid (USA samples)	Stronger cytoplasmic p-Akt in tumors compared with nuclear and cytoplasmic staining in normal. Greatly increased nuclear mTOR in the tumors	Liu and Brown (58)
**p-Akt(Ser473)** *CST 1:50*	83 PTC (samples from Greece)	Trend for increased expression in tumors with aggressive features. High levels in tumors with *PIK3CA* and *PTEN* mutations	Sozopoulos et al. (59)
**p-Akt(Ser473)** *CST 1:50*	35 PTC; 16 with associated lymph node metastasis compared with 19 without (samples from Spain)	No difference in p-Akt staining between tumors with associated metastasis and those without	Zafón et al.^a^ (60)
**p-Akt(Ser473)** *CST*	25 PTC	Staining greater at the invasive front and cells infiltrating vasculature. Associated with connexin 43.	Jensen et al. (53)
	10 FTC (samples from Finland)		
**p-Akt(Ser473)** *CST*	30 PTC (15 classical and 15 histological variants) (USA samples)	Low to moderate staining for p-Akt in all cancers. Enhanced nuclear p-mTOR in aggressive variants	Liu and Brown (61)
**p-Akt(Ser473)** *Rabbit polyclonal CST*	536 PTC on TMA (Saudi Arabian cohort)	Of 446 informative TMA results, 242 (54.3%) were classified as high p-Akt and 204 (45.7%) as low (Table 2 in Supplementary Material of the paper); highly significant correlation with mTORC2 activity as measured using mTOR(Ser2482) antibody staining (*P* < 0.0006)	Ahmed et al. (62)
**p-Akt(Ser473)** *Novacastra NCL-L-Akt-Phos 1:40*	12 ATC (samples from Serbia)	High p-Akt in 5/12 (41.6%), high p-ERK in 7/12 (58.3%), low level PTEN in 6/12	Milosevic et al. (63)
		Significant negative correlation between p-Akt staining and *NRAS* gene mutations/p-ERK staining	
**p-Akt(Ser473)** *CST*	42 tumor samples (5 FTC,10 conventional PTC, 8 aggressive PTC, 3 poorly differentiated classified as PTC; 16 MTC) (samples from Greece)	Strong positive association between p-Akt(Ser473) and Sin1 protein expression (all 42 tumors combined)	Moraitis et al. (64)
**p-Akt(Ser473)** *CST Rabbit polyclonal* (*9271*)	10 normal thyroid	Overexpression in 57% of PTC, and 30% of FTC. FVTC did not overexpress p-Akt	Pringle et al. (39)
	30 PTC		
	5 FVTC		
	10 FTC (samples from Ukraine)		
**p-Akt(Ser473)** *CST*	1022 PTC on TMA (Saudi Arabian cohort)	Paper focused on XIAP. Highly significant association between XIAP staining and p-Akt staining	Hussain et al. (65)
**Medullary thyroid cancers (tumours arising from C cells)**			
**p-Akt(Ser473)** *Rabbit polyclonal CST* (*736E11*) *1:1000*	TMA	38/49 showed positive staining	Rapa et al. (66)
	49 MTC (samples from Italy)	Associated with p-mTOR and p-p70S6K, but not with clinical features, pathological features, prognosis, or RET mutation status	
**p-Akt(Ser473)** *Rabbit polyclonal CST* (*736E11*) *1:50*	TMA	Expressed in all tumors, but mostly weak. No significant association with patient outcome	Erovic et al. (67)
	23 MTC		
	Normal thyroid control (USA cohort)		
**p-Akt (Ser473)** *CST1:50* and **p-Akt (Thr308)** *CST1:25*	TMA 18 hereditary MTC 35 sporadic MTC 37 C cell-free thyroid tissue Tissue sections 20 MTC (USA cohort)	C cell-free thyroid tissue negative for both p-Akt(Ser473) and p-Akt(308) 12/21 (43%) primary MTCs positive for p-Akt(Thr308) 1/1 LNM positive for p-Akt(Thr308) 36/46 (78%) primary MTCs positive for p-Akt(Ser473) 15/19 (79%) LNM positive for p-Akt(Ser473) (see Table S2 in Supplementary Material of the paper)	Tamburrino et al. (68)

*^a^Article in Spanish*.

*CST, Cell Signaling Technology; mAb, monoclonal antibody; FA, follicular adenoma; PTC, papillary thyroid carcinoma; FTC, follicular thyroid carcinoma; ATC, anaplastic thyroid carcinoma; MTC, medullary thyroid carcinoma; HT, Hashimoto’s thyroditis, FVPC, follicular variant papillary carcinoma; TMA, tissue microarray; LNM, lymph node metastasis*.

Liu et al. (55) observed p-Akt(Ser473) immunoreactivity in 16/25 follicular thyroid cancers, and found genetic alterations in the PI3K/Akt pathway to account for all of these, including copy gains of *PIK3CA*, *PIK3CB*, and *AKT2*. In anaplastic cancers 16/27 showed p-Akt(Ser473) immunoreactivity. The vast majority (95.8%) of anaplastic thyroid cancers had genetic alterations resulting in activation of both the PI3K and MAPK pathways, including frequent co-existence of receptor tyrosine kinase copy number gain and gene mutation, as well as *PIK3CA*, *PIK3CB*, and *PDK1* copy number gains, *PTEN* mutation and *BRAF* mutation. Furthermore, RAS activation can result in activation of the PI3K/Akt pathway in thyroid carcinoma (69). However, a recent study of 12 anaplastic carcinomas reported that p-Akt(Ser473) staining inversely correlated with p-ERK staining and *NRAS* gene mutations, perhaps suggesting that only one of these two signaling pathways might drive progression to anaplastic tumors (63).

Overexpression of upstream receptors can elevate levels of p-Akt in thyroid cancers. Liu et al. (55) found that *EGFR* and *VEGF1* copy number gain was particularly common in both follicular and anaplastic thyroid cancer. Rearranged during transfection (*RET*) is a tyrosine kinase receptor proto-oncogene, point mutation of which underlies Multiple Endocrine Neoplasia Types 2A and 2B (MEN2A/MEN2B) and familial medullary thyroid cancer. Some papillary thyroid carcinomas, including those arising after ionizing radiation exposure, develop through translocations resulting in fusion of *RET* with 1 of 13 other genes (*RET/PTC* rearrangements) (70) producing a protein with constitutively active tyrosine kinase signaling. There is evidence to show that a MEN2A-type mutant *RET* results in constitutive activation of the PI3K/Akt pathway (71), and papillary cancers with RET overexpression due to gene rearrangements demonstrated the greatest p-Akt levels and throughout the tumors (48). c-Met, the hepatocyte growth factor receptor, was associated with p-Akt expression in papillary thyroid carcinomas in a Middle Eastern population in which *PIK3CA* mutation was rare (54). The tyrosine kinase receptor EphA2 is overexpressed in benign and malignant thyroid tumors and has been shown to contribute to p-Akt activation (72).

Downstream targets have been associated with high levels of p-Akt(Ser473). The majority of thyroid cancers exhibit reduced nuclear FoxO3A (10), and FoxO3A downstream target genes *CDKN1B* encoding cyclin-dependent kinase inhibitor p27^KIP1^, and *BCL2L11* encoding Bim, a pro-apoptotic member of the Bcl-2 family of proteins, have been identified as functionally relevant in follicular rat thyroid cells (10) (Figure 1). Inhibition of PI3K/Akt signaling by LY294002 resulted in reduced FoxO3A phosphorylation and its accumulation in the nucleus, and increased *p27*^*KIP1*^ and *Bim* mRNA *in vitro*. Vasco et al. (48) reported that invasive regions of papillary and thyroid cancer were characterized by both nuclear p-Akt and nuclear exclusion of p27^KIP1^ and used a human papillary thyroid cancer cell line (NPA) to link p-Akt and cytoplasmic exclusion of p27^KIP1^ with invasive capacity in a transwell assay. In addition, Bad and XIAP have been noted as relevant Akt targets in papillary thyroid carcinoma cells (62, 65), contributing to protection from apoptosis.

Enhanced nuclear p-mTOR suggests the activation of the PI3K/Akt/mTOR pathway in aggressive variants of papillary thyroid carcinomas (61). The Al-Kuraya group have performed tissue microarray immunohistochemistry for p-Akt(Ser473) and related proteins on a large series of papillary thyroid carcinomas of Middle Eastern origin, enabling correlative expression studies to be performed (albeit with some loss of informative spots on different staining runs). In a recent study, p-mTOR(Ser2448) was used as a marker of mTORC1 activity and was detected in 81% of tumors, while p-mTOR(Ser2481), a marker for mTORC2 activity was observed in 39%. There was a strong positive correlation with p-Akt(Ser473) and p-mTOR(Ser2481) staining, as well as activation of further Akt and mTORC1 target proteins. As evidence for the concept that both mTOR complexes are actively involved in regulating the survival of papillary thyroid carcinoma cells, Torin 2, a dual mTORC1/2 inhibitor, induced apoptosis in two papillary carcinoma cell lines, BCPAP and TPC, in association with reduced activity of Akt and downstream targets of p-Akt and mTOR complexes (62).

Strong positive association has been observed between staining for p-Akt(Ser473) and Sin1 protein, a component of the mTORC2 complex, in a series of 42 tumor samples, which included 16 medullary thyroid cancers (see below). This was a characteristic of aggressive papillary and medullary carcinomas and therefore further staining of larger cohorts for p-Akt in conjunction with Sin1 is worthwhile, since normal thyroid tissues showed no expression or <10% weakly Sin1 positive cells (64). In these cells, S6K appeared to be the relevant Sin1 kinase. In a series of 58 ovarian cancer specimens, p-Akt(Ser473) and p-Sin1(Thr86) staining were, if anything, negatively correlated, but this did not reach statistical significance. The generation of the phospho-specific Sin-1 antibodies, along with the discovery that mutation of the Sin1 phosphorylation sites occurs naturally in ovarian and skin cancers and abrogates the negative control on Akt activity (73), could be helpful in determining the mechanisms for the positive association between p-Akt(Ser473) and Sin1 in thyroid cancers.

Whether p-Akt(Ser473) expression is associated with actual patient prognosis has yet to be measured in thyroid cancer, and only inferences can be made from the literature. p-Akt(Ser473) was not associated with likely prognosis of papillary carcinoma (59). In a more recent study, the Al-Kuraya group stained for the anti-apoptotic protein XIAP on tissue microarray. Of 989 tumors producing informative staining for p-Akt(Ser473), 57.1% exhibited staining classed as high, and there was a highly significant positive association with staining for XIAP (65). XIAP staining was associated with poor disease free survival, but the study did not report on p-Akt directly in relation to prognosis.

Patterns of p-Akt staining have been reported to vary between follicular and papillary carcinomas, but more prominent nuclear p-Akt(Ser473) was reported in regions of invasion in both types (48). Similarly, while differentiated thyroid cancers demonstrate cytoplasmic staining, contiguous anaplastic thyroid cancers display both nuclear and cytoplasmic p-Akt(Ser473) (56). These observations may suggest a role of nuclear p-Akt in aggressive or invasive disease. This hypothesis is supported by findings that nuclear p-Akt is associated with migration *in vitro* (48), and that in the TRPV mouse model, p-Akt is both cytoplasmic and nuclear in primary tumors, but is predominantly nuclear in metastases (38). By contrast to these studies, Zafón et al. (60) did not find any difference in cytoplasmic or nuclear p-Akt(Ser473) staining between papillary thyroid cancers with or without associated lymph node metastases at the time of diagnosis, even though the group with associated metastases had a greater proportion overexpressing RET or EGFR.

## Medullary Thyroid Cancer

p-Akt(Ser473) expression has also been investigated in medullary thyroid cancer, which unlike other subtypes of thyroid cancer is derived from parafollicular C-cells. Evidence from hereditary cancers and *in vitro* work suggests that the PI3K/Akt pathway may play a role in medullary thyroid carcinogenesis (74). Only three studies have so far reported p-Akt staining in human medullary thyroid cancers, and these used tissue microarrays (Table 1). p-Akt(Ser473) expression was strongly associated with p-mTOR and p-p70S6K in a series of 49 cancers, suggesting that the Akt/mTOR pathway is active in these tumors, and is therefore potentially amenable to therapy (66), but there was no association between p-Akt expression and prognosis or clinicopathological factors (66). Furthermore, in a study of 23 cancers, p-Akt(Ser473) and mTOR staining were predominantly weak, with no association between p-Akt(Ser473) and overall or disease-free survival (67). However, Tamburrino et al. (68) found significant correlations between p-Akt(Ser473) and p-ERK staining and Pennelli et al. (75) also remarked on the preferential activation of the PI3K/Akt pathway, detected as high levels of p-Akt(Ser473) on western blotting in medullary thyroid carcinoma cases which had mutant *RAS*.

In the Tamburrino study (68), a series of 53 human medullary thyroid carcinoma tissues (18 hereditary, 35 sporadic), ribosomal protein p-S6(Ser235/236) was frequently overexpressed compared to normal thyroid tissues. It should be noted that the normal thyroid used was largely C cell-free, and therefore the malignant cells were not being compared with their normal counterparts; however, other reports make no mention of positive C-cells in control normal thyroid tissue, which is classed as negative. Of the medullary tumors, 76% had detectable p-Akt(Ser473) and 96% expressed detectable levels of S6K. That S6K is more frequently detected than p-Akt is similar to findings of Pringle et al. (39) in their mouse model of follicular thyroid carcinoma. By contrast to p-Akt(Ser473), only 39% of tumors had detectable p-Akt(Thr308), perhaps due to difference in the sensitivity of the antibodies, as suggested by the authors, although far fewer tumors were stained for p-Akt(Thr308) than p-Akt(Ser473). A further relevant Akt target in medullary thyroid carcinoma may be the tumor suppressor, Programed Cell Death 4 (PDCD4), which Akt inactivates, which showed reduced expression in medullary thyroid carcinoma (75). Overall, immunohistochemistry results support previous observations of elevated Akt/mTOR signaling in medullary thyroid carcinoma. Furthermore, rapamycin reduced tumor volume of RET mutant MZ-CRC-1 xenografts in NOD/SCID mice (68).

## Parathyroid Carcinoma

Parathyroid carcinomas are rare, accounting for <1% of primary hyperparathyroidism (76). These cancers are aggressive, with high risk of metastasis and recurrence. Parathyroid cancers remain poorly understood, and surgery remains the mainstay of treatment; little is known about the role of PI3K/Akt signaling in these tumors. p-Akt expression has been described in a cohort of 10 parathyroid carcinomas and 25 parathyroid adenomas (Table 2). In this study, p-Akt expression was reported to be variable; however, expression appeared to be lower in carcinomas: on average 28% of tumor cells were positive in carcinomas, with 87% positive in adenomas, although no statistical analysis was presented (77). p-mTOR and FoxO1 expression were also examined in this cohort, and the authors suggested that there was no marked alteration in their expression in parathyroid carcinoma compared to adenoma (77), but the exact picture remains unclear. The PI3K/Akt pathway has, to the best of our knowledge, remained otherwise unstudied in parathyroid neoplasia. However, mutation analysis from a single patient has identified an activating mutation in *PIK3CA* from the primary tumor, and RNA-seq datasets demonstrated overexpression of *AKT2* mRNA in one of two areas of recurrence (78), perhaps suggesting that this pathway may be an important area for future research.

**Table 2:** **Results of Immunohistochemical staining studies for p-Akt(Ser473) in non-thyroid tumors of endocrine tissue origin**.

Antibody	Specimens	Key results	Author (reference)
**Parathyroid carcinoma**			
**p-Akt(Ser473)** *CST, 736E11, 1:50*	10 parathyroid carcinoma	On average 87% of tumor cells were positive in adenomas, and 28% of tumor cells were positive in carcinomas	Erovic et al. (77)
	25 parathyroid adenoma (Canadian Specimens)		
**Tumours of the pituitary**			
**p-Akt(Ser473)** *CST, Rabbit Polyclonal, 1:50*	40 pituitary adenoma (*ACTHoma 10, GHoma 10, PRLoma 10, NFPA 10*)	Increased expression compared with normal pituitary. No correlation with p27^KIP1^ expression	Musat et al. (79)
	10 Normal pituitary		
**p-Akt (unspecified)***SC, 1:100*	19 ACTHoma (Japanese Specimens)	Correlation between expression of p-Akt and PC2	Iino et al. (80)
**p-Akt(Ser473)** *CST, D9E, 1:50*	35 incompletely resected NFPA	Expression associated with recurrence (sensitivity: 69.2%, specificity: 66.7%)	Noh et al. (81)
**p-Akt(Ser473)***CST, D9E 1:100*	30 pituitary adenoma (*GHoma 7, PRLoma 6, ACTHoma 4, FSHoma 1, NFPA 12*) (Italian Specimens)	p-Akt correlated with c-met and HGF, but not PI3K. Endothelial p-Akt expression associated with tumor size	Trovato et al. (82)
**Tumours of the adrenal cortex**			
**p-Akt(Ser473)** *CST*	4 adrenocortical carcinoma	p-Akt detectable in normal adrenal cortex. Focal expression of p-Akt in adrenocortical carcinoma	Fassnacht et al. (83)
	2 normal adrenal (from renal cancer surgery) (German Specimens)		
**p-Akt(Ser473)***ILT*	24 adrenocortical carcinoma	Adrenocortical carcinoma showed increased expression of p-Akt compared with adenomas or normal tissue, along with increased p-IGF1R	Barlaskar et al. (84)
	2 adrenocortical adenoma		
	4 normal		
**p-Akt (Ser473)** *CST*	121 adrenocortical carcinoma	p-Akt expression not detected in normal adrenal or adrenocortical adenoma, but detected in 33% of adrenocortical carcinoma. Low SGK1 with strong p-Akt associated with poor prognosis. No correlation between p-Akt and nuclear β-catenin staining	Ronchi et al. (85)
	15 adrenocortical adenoma		
	5 normal adrenal		
**p-Akt (Ser473)** *CST Rabbit mAb, D9E, 1:25*	47 adrenocortical carcinoma (Dutch Specimens)	p-Akt expression associated with better prognosis after mitotane monotherapy	Hermsen et al. (86)
**Tumours of the adrenal medulla – neuroblastoma**			
**p-Akt(Ser473)** *CST*	4 adrenocortical carcinoma	p-Akt detectable in normal adrenal cortex. Focal expression of p-Akt in adrenocortical carcinoma	Fassnacht et al. (83)
	2 normal adrenal (from renal cancer surgery) (German Specimens)		
**p-Akt(Ser473)***CST, 1:400*	24 neuroblastoma	No significant difference between differentiated and undifferentiated tumors	Qiao et al. (87)
**p-Akt(Ser473)** *Rabbit mAb, 1;100, CST*	116 neuroblastoma (German Specimens)	p-Akt(Ser473) and/or p-Akt(Thr308) expression in majority of tumors. High expression correlated with *MYCN* amplification or 1p36. Expression of p-Akt(Thr308) or expression of both isoforms associated with stage IV disease and worse prognosis	Opel et al. (88)
**p-Atk(Thr308)** *Rabbit mAb, 1:100, CST*			
**p-Akt(Ser473)** *CST*	30 neuroblastoma	Cytoplasmic p-Akt expression neuroblastoma. Negative staining in normal medulla	Johnsen et al. (89)
**p-Akt(Ser473)** *SC, polyclonal rabbit, 1:100*	55 mass screening neuroblastoma, 21 paired metastasis (Japan, Canada)	Expression lower in neuroblastoma from mass screening, and lower in younger patients. Expression correlates with PARP-1	Sartelet et al. (90)
	55 matched unscreened neuroblastoma, 21 paired metastasis (France)		
**p-Akt(Ser473)** *SC, S473-r, 1:100*	101 primary neuroblastoma, 39 metastasis (French specimens)	Expression in majority of tumors, and expression correlation with PI3K, Akt, VEGFR1, VEGF, TRKB and IGF1R. Not associated with survival	Sartelet et al. (91)
**p-Akt(Ser473)** *SC S473-r, rabbit polyclonal, 1:100*	280 primary neuroblastoma, 97 metastasis (French and Canadian specimens)	Expression in almost all tumors. Expression higher in CD133^+^ tumors	Sartelet et al. (92)
**Tumours of adrenal medulla – pheochromocytoma**			
**p-Akt(Ser473)** *CST*	8 phaeochromocytoma	Increased expression compared to normal adrenal medulla	Fassnacht et al. (83)
	2 normal adrenal (from renal cancer surgery) (German Specimens)		
**p-Akt(Ser473)** *CST, 736E11, 1:50*	39 primary and 8 metastatic pheochromocytoma	Trend for increased levels in primary tumors versus normal medulla, and in metastasis versus primary tumors	Chaux et al. (93)
	19 normal adrenal medulla (American Specimens)		
**Gastroenteropancreatic neuroendocrine tumours**			
**p-Akt(Ser473)** *Abcam, ab89232, 1:100*	85 gastroenteropancreatic NET	Cytoplasmic staining in more than 70% of tumors, vascular endothelium also positive. p-Akt expression correlated with p-EGFR and p-ERK1/2	Shah et al. (94)
	5 paraganglioma		
	4 NET of unknown origin		
**p-Akt(Ser473)***CST, 587F11, 1:100, Mouse mAb*	46 gastroenteropancreatic NET (American Specimens)	Diffusely granular cytoplasmic staining in 61% of tumors. No correlation with grade, size or metastasis	Ghayouri et al. (95)
**p-Akt(Ser473)** *CST, 736E11, 1:50 Rabbit mAb*	20 gastroenteropancreatic NET (Japanese Specimens)	Cytoplasmic and nuclear p-Akt staining, co-expression with p-mTOR in some tumor cells	Shida et al. (96)
**p-Akt(Ser473)** *CST*	25 neuroendocrine carcinoma (carcinoid any site or pancreatic NET; treated with everolimus and octreotide)	p-Akt(Ser473) immunostaining of archival tumor blocks not associated progression-free survival. High p-Akt(Thr308) from pre-treatment FNA measured using RPPA associated with longer PFS	Meric-Bernstam et al. (97)
**p-Akt(Ser473)** *CST*	195 NET (124 small intestinal, 14 pancreatic, 52 others)	Correlations between members of the mTOR pathway. p-Akt expression correlated with expression of p-PDPK1 and p-mTOR, but was not associated with prognosis	Qian et al. (98)

*CST, Cell Signaling Technology; SC, Santa Cruz Biotechnology Inc.; ILT, Invitrogen Life Technologies; mAb, monoclonal antibody; GHoma, somatotroph adenomas (growth hormone secreting); ACTHoma, corticotroph adenomas (adrenocorticotrophic hormone secreting); TSHoma, thyrotroph adenomas (thyroid-stimulating hormone secreting); FSHoma, follicle-stimulating hormone secreting; NFPA, Non-functioning pituitary adenomas; NET, neuroendocrine tumors; HGF, hepatocyte growth factor; SGK1, serum GC kinase 1; VEGFR1, vascular endothelial growth factor receptor-1; TRKB, neurotrophic tyrosine kinase receptor type B; p-IGF1R, phosphorylated insulin-like growth factor 1 receptor; p-EGFR, phosphorylated epidermal growth factor receptor; p-PDPK1, phosphorylated phosphoinositide-dependent kinase-1; FNA, fine needle aspirates; RPPA, Reverse Phase Protein Array; PFS, progression-free survival*.

## Pituitary Tumors

Pituitary adenomas are among the most common intracranial neoplasms. Approximately 60% of pituitary tumors are functionally active, of which prolactinomas [prolactin (PRL) secreting tumors] are the most common subtype. The remaining functional tumors are somatotroph adenomas [growth hormone (GH) secreting], corticotroph adenomas [adrenocorticotrophic hormone (ACTH) secreting], thyrotroph adenomas [thyroid-stimulating hormone (TSH) secreting], and gonadotroph adenomas [follicle-stimulating hormone (FSH) and luteinizing hormone (LH) secreting]. Non-functioning pituitary adenomas (NFPA) do not exhibit hypersecretion. Of note, some pituitary adenomas are endocrinologically “silent”: they produce hormones that are not secreted (99). Malignancy is rare, however, local invasion, recurrence, or treatment failure occur in a proportion of cases.

The PI3K/Akt pathway plays a role in maintaining viability of rat pituitary adenoma cell lines (GH3, LβT2) (100, 101), suggesting a potential role for Akt in pituitary tumorigenesis. This is further supported by work in *TR*β^PV/PV^ mice and MENX (multiple endocrine neoplasia-like syndrome) rats. *TR*β^PV/PV^ mice possess a knock-in mutation of the TRβ gene and spontaneously develop TSH-secreting pituitary adenomas (TSHoma) with age (102); MENX rats harbor biallelic frameshift mutations of the *CDKN1B* gene (encoding p27^KIP1^) and develop pituitary adenomas alongside multiple other endocrine tumors (103). Pituitary adenomas from *TR*β^PV/PV^ mice show increased phosphorylation of Akt (Ser473), mTOR and S6 on western blotting, and PI3K/Akt inhibition decreases pituitary tumor mass and increases apoptosis (102). Likewise, immunohistochemistry demonstrated increased levels of p-Akt(Ser473) in pituitary adenomas from MENX rats, and increased p-Akt(Ser473), p-Akt(Thr308), and p-S6(Ser24/44) in primary cultures of tumor cells (104). The MENX rat model has been useful in demonstrating anti-proliferative and pro-apoptotic function of the dual PI3K/mTOR inhibitor NVP-BEZ235 *in vivo* and in organotypic culture (105).

Compared with normal tissue, human pituitary adenomas showed an increased percentage of cells immunostaining for p-Akt(Ser473) on immunohistochemistry, and increased p-Akt:total Akt ratios on western blotting suggest that Akt phosphorylation is increased in these tumors (79). Likewise, in a study of three gonadotropinomas, levels of p-Akt(Thr308) were elevated in protein lysates of tumors versus normal pituitaries (101), but to our knowledge there have been no larger studies examining Akt phosphorylation at Thr308 in human pituitary tumors. Few studies have investigated the prognostic role of p-Akt in pituitary tumors. However, in partially resected NFPA, p-Akt(Ser473) immunostaining was associated with tumor recurrence, suggesting that Akt phosphorylation may be of use in risk stratification in these cases (81). A recent study has also revealed that endothelial expression of p-Akt(Ser473) in pituitary tumors is positively associated with tumor size, leading the authors to hypothesize that Akt activation may play a role in angiogenesis in these tumors (82). A study of ACTH-producing pituitary adenomas has revealed significant associations between the immunostaining for p-Akt(Ser473) and prohormone convertase 2 (a serine protease involved in proteolytic cleavage of prohormones), but the relevance and implications of these findings remain unclear (80).

The mechanism for Akt over-activation in pituitary tumors is likely to be multifactorial. *PIK3CA* amplifications or mutations occur in around a third of pituitary tumors (106, 107), and *PIK3CA* mutations are associated with invasive disease (106). It is well established that there is altered or overexpression of growth factors and growth factor receptors in pituitary tumors, for example alterations in epidermal growth factor receptor (EGFR) and fibroblast growth factor receptor (FGFR) signaling, which could also drive Akt activation [reviewed in Ref. (108)]. In particular, Eps8 (Epidermal Growth Factor Receptor Pathway Substrate 8), which signals downstream of these receptors, promoted cell survival through modulation of Akt activity in Eps8-overexpressing LβT2 gonadotrope cells *in vitro*, and immunoblotting demonstrated that both p-Akt(Thr308) and Eps8 are overexpressed in human pituitary adenomas (101). Hepatocyte growth factor receptor (HGFR/c-met) signaling may act as a further driver for Akt activation: immunohistochemistry has demonstrated significant association between p-Akt(Ser473), c-met and HGF in human pituitary adenomas (82). The role for PTEN is less clear: while no *PTEN* mutations were identified in 33 pituitary tumors, and *PTEN* mRNA levels were comparable to normal tissue, nuclear PTEN expression was reduced in pituitary tumors (79). Investigations are needed to determine whether this results in activation of nuclear Akt pools. Interestingly, it has also been suggested that altered levels of microRNAs may play a role in pituitary tumorigenesis by modulating PI3K/Akt signaling via regulation of PTEN expression (109).

Downstream PI3K/Akt signaling in human pituitary tumors remains relatively poorly understood. Nuclear p27^KIP1^ is reduced in human pituitary adenomas, potentially consistent with cytoplasmic sequestration of p27^KIP1^ by Akt activity; however, the lack of correlation between p-Akt(Ser473) and p27^KIP1^ expression means conclusions must remain cautious (79). Pituitary adenomas have also been reported to have increased levels of mTOR activation, as measured by phosphorylation of downstream S6, with GHomas showing the most marked elevation of mTOR activity (110). However, mTOR activity appeared to be independent of PI3K/Akt signaling in pituitary adenoma cells on primary culture, and mTOR activity was not related to clinicopathological characteristics in the sample of 53 pituitary adenomas (110). Increased Cyclin D1 expression has been consistently reported in non-functioning pituitary tumors (111, 112). This is partially due to Cyclin D1 (*CCND1*) gene amplification, which occurs in around 25% of pituitary adenomas (111). Overexpression of Cyclin D1 in tumors without gene amplification may suggest upstream pathways such as PI3K/Akt signaling (112). However, although reduced levels of double phosphorylated c-Myc (Thr62/Ser58) are potentially consistent with activation of the PI3K/Akt pathway, phosphorylated and total TSC2 and mTOR levels were unchanged (112).

Targeting PI3K/Akt/mTOR signaling is of potential therapeutic interest in human pituitary tumors. mTOR inhibition reduces viability of human pituitary tumor cells *in vitro* (113). Interestingly, in human NFPA cells, a response to everolimus was more likely in cells originating from younger patients, of female gender, with invasive macroadenomas (113). As in other cell types, mTOR inhibition induces Akt activation in pituitary cells *in vitro*, and this may contribute to resistance to mTOR inhibitors, which may limit their clinical efficacy (114). Therefore, concomitantly targeting the upstream PI3K/Akt pathway is potentially promising: combining rapamycin with octreotide (a somatostatin analog) more effectively suppresses pituitary tumor cell proliferation than rapamycin alone, with combination treatment proving effective in NFPA cells resistant to single agent rapamycin (114). Likewise, in primary cultures of pituitary tumor cells from MENX rats, cell viability is more effectively reduced by the dual PI3K/mTOR inhibitor NVP-BEZ235 than by everolimus alone (104). Targeting the PI3K/Akt/mTOR pathway could also be combined with more traditional treatment modalities in pituitary tumors, potentially increasing efficacy and/or reducing toxicity by allowing dose reduction: mTOR inhibitors increase radiosensitivity of pituitary tumor cells *in vitro* (115), and temozolomide and XL765 (a dual PI3K/mTOR inhibitor) synergistically inhibit cellular proliferation *in vitro* and reduce tumor growth and hormone secretion in xenograft mice (116). Despite the promising preclinical work, experience in human pituitary tumors is limited to an isolated report of unsuccessful treatment with everolimus in a single patient with a pituitary carcinoma (a rare entity defined by presence of metastasis), and as such, no clear conclusions can be made about efficacy of PI3K/Akt/mTOR inhibition in human pituitary tumors (117).

## Adrenal Cortex

Most adrenocortical tumors are sporadic, but adenomas or carcinomas may occur in association with familial cancer syndromes (118). Adrenocortical carcinomas (ACC) are generally highly aggressive tumors, with an incidence of approximately 1–2 cases per million per year (119). Complete surgical resection is potentially curative; however, recurrence is relatively common. Treatment of advanced disease remains challenging: mitotane plus etoposide, doxorubicin and cisplatin is the first-line cytotoxic therapy but response rate is relatively poor (119). The pathophysiology of these rare tumors remains poorly understood, but a greater understanding of key signaling pathways may provide much-needed treatment options.

Evidence suggests that IGF signaling may be of great clinical interest in ACC. Patients with Beckwith–Wiedemann Syndrome, in whom deregulated imprinting at 11p15.5 leads to biallelic expression of *IGF2*, have a 5% incidence of ACC (118). Furthermore, *IGF2* is the most overexpressed gene in a large proportion of sporadic ACC and on transcriptome analysis forms part of a cluster of eight overexpressed genes characterizing malignant ACC rather than prevalent adrenocortical adenomas (ACA) (120). Early clinical trials of IGF-1R inhibitors showed potentially promising results in these rare cancers (121, 122), but unfortunately, the IGF-1R and insulin receptor inhibitor linsitinib showed limited therapeutic benefit in a recent phase III trial (123). Nonetheless, the majority of these tumors possess overactivation of the IGF pathway and/or enhanced EGFR signaling (124, 125), which could serve as further driver of Akt activation. Altered β-catenin expression may also regulate Akt activation in these tumors: β-catenin expression is known to be altered in 20% of ACC, and knockdown of β-catenin in human ACC cells *in vitro* decreases Akt phosphorylation (126), although no correlation was seen between p-Akt(Ser473) and β-catenin immunostaining in human tumors (85).

Akt activation appears to be enhanced in a subset of human ACC *in vivo* (Table 2). An early study by the Allolio group showed that total Akt and p-Akt(Ser473) were detectable in the adrenal cortex but not in the medulla, and ACC exhibited focal enhancement of p-Akt(Ser473), potentially suggesting local activation of Akt signaling in these cancers (83). The results from western blotting did not necessarily correlate with results obtained by immunohistochemistry, but this perhaps reflected the focal expression of p-Akt and also areas of stromal and necrotic tissue which are both negative for total Akt and p-Akt in ACC (83). A more recent study found elevation of both total Akt and p-Akt(Ser473) in six adrenocortical adenomas and four ACCs compared to normal adrenocortical controls on western blotting (127). Nakamura et al. (128) also detected p-Akt(Ser473) in the adrenal cortex in their immunohistochemical study, but comparing ACC (41 samples) with ACA (54 samples) there was a similar percentage of p-Akt(Ser473) positive cells. By contrast, another group demonstrated increased p-Akt(Ser473) expression in 24 ACC compared with 22 ACA or 4 normal tissue samples (84). Likewise, a more recent study of 121 ACC from the Allolio group describes enhanced p-Akt(Ser473) immunostaining in one-third of carcinomas, which was not seen in ACA or normal tissue (85).

The association between p-Akt(Ser473) expression and survival in ACC remains unclear. In one study, p-Akt(Ser473) expression alone was not associated with prognosis, but high p-Akt and low SGK1 (Serum/Glucocorticoid Regulated Kinase 1) expression identified a subgroup with extremely poor prognosis (85). In contrast, in tissue microarrays of 45 ACC patients who received mitotane monotherapy, in which p-Akt was detected in 31% of tumors, Hermsen et al. (86) unexpectedly found an association between p-Akt expression and longer overall survival, although there was no association with objective response to mitotane.

Early results suggest that targeting p-Akt signaling may be a promising therapeutic strategy in ACC. mTOR inhibitors sirolimus and temsirolimus inhibit ACC cell proliferation *in vitro* (129). Likewise, everolimus treatment results in antitumor effects in primary adrenocortical cultures, ACC cell lines, and in xenograft mice (130). Of note, significant synergism is seen when everolimus is combined with sorafenib (a tyrosine kinase inhibitor), suggesting that targeting signaling pathways at multiple levels may be of value in these tumors (130). Moreover, the dual PI3K/mTOR inhibitor, NVP-BEZ235 inhibits Akt phosphorylation, reduces ACC cell proliferation *in vitro*, and reduces tumor size in xenograft mice (131). The H295 cell line overproduces IGF2, which likely stimulates IGF-1R in an autocrine signaling loop, and this provided a useful model of the common IGF2 overproduction in ACC; IGF2-neutralizing antibody increased the anti-proliferative effects of sirolimus (129). Encouragingly, the combination of an IGF-1R inhibitor and mTOR inhibition with cixutumumab (monoclonal antibody targeting IGF-1R) and temsirolimus resulted in 42% of patients with ACC achieving stable disease of at least 6 months (132).

## Adrenal Medulla: Phaeochromocytoma and Paraganglioma

Phaeochromocytomas and paragangliomas are catecholamine-secreting neuroendocrine tumors of chromaffin cells, either of the adrenal medulla (phaeochromocytoma) or extra-adrenal sympathetic or parasympathetic paraganglia (paraganglioma). Most phaeochromocytomas are apparently sporadic, although a significant proportion is associated with genetic syndromes, such as MEN2A, Neurofibromatosis (NF1), Von-Hippel Lindau (VHL), or germline mutations in *SDHx* (133). In the vast majority of cases, the disease is benign and surgical excision is curative. While estimates vary, approximately 10% are malignant, and in these cases current treatment options remain limited (134, 135).

*In vitro* evidence suggests that the PI3K/Akt pathway is of interest in phaeochromocytoma. It is well established that Akt signaling promotes survival of rat PC12 pheochromocytoma cells and neuronal cells *in vitro*, protecting against apoptosis induced by a range of stimuli, including cytotoxic chemotherapy (136–139). Furthermore, PI3K/Akt signaling may promote the neuroendocrine phenotype as inhibition of PI3K/Akt in PC12 cells reduces neuroendocrine marker expression and hormone secretion (139). PI3K/Akt signaling is also essential for anchorage-independent growth in rat fibroblasts expressing *RET-MEN2A* (71); *RET-MEN2A* germline mutations are responsible for MEN2A, which leads to pheochromocytoma in approximately 50% of patients – therefore these results suggest that this pathway may be critical in tumorigenesis in these patients.

Mice haplodeficient for *PTEN* develop bilateral tumors of the adrenal medulla with high penetrance; concomitant deletion of *AKT1* (*PTEN*^+/−^*AKT1^−/−^*) in these rodents significantly reduces the size of the adrenal medulla and the tumors, and reduces the occurrence of high grade lesions, suggesting that Akt signaling plays a role at least in advanced tumors in these mice (34). However, *PTEN* mutation does not appear to be a major driver for Akt activation in human phaeochromocytomas, as *PTEN* mutations are reported to be rare in human tumors (83). A second rodent model, produced by ectopic overexpression of *ErbB-2* in mice, leads to catecholamine-producing adrenal tumors with greatly reduced PTEN protein levels, and increased Akt phosphorylation and cyclin D1 (140). In this mouse model, while *PTEN* mRNA levels were somewhat reduced, this reduction did not appear to account for the marked reduction in protein levels, leading the authors to suggest that post-translational regulation of PTEN may play an important role (140). MENX rats represent a third rodent model of pheochromocytoma; these rats possess homozygous frameshift mutations in *CDKN1B* and develop tumors of the adrenal medulla alongside other endocrine tumors (141). Adrenal tumors of MENX rats overexpress the secretin receptor, and PI3K/Akt signaling acts downstream to promote cell proliferation (141). However, this pathway does not appear to be directly applicable to humans, as human phaeochromocytoma rarely overexpress the secretin receptor (141). MENX pheochromocytomas also overexpress secreted BMP7 (Bone Morphogenic Protein 7); this parallels elevated immunoreactivity for BMP7 in human pheochromocytoma samples, detected in 72% of 184 tumor samples on tissue microarray (150 pheochromocytomas and 34 paragangliomas) (142), and BMP7 knockdown was demonstrated to promote proliferation, migration, and invasion primary cultures of MENX-derived pheochromocytomas. From *in vitro* models, including PC12 cells transfected to overexpress BMP7, evidence was provided for non-canoncial signaling through the PI3K/Akt/mTOR pathway leading to overexpression of integrin β1, responsible for the BMP7-induced cancer phenotypes (142).

Two separate studies have shown elevated levels of p-Akt(Ser473) in human phaeochromocytoma using western blotting (83, 139). In one study, expression of p-Akt(Ser473) was further categorized in a smaller sample of human phaeochromocytoma using immunohistochemistry, demonstrating minimal or absent immunostaining in normal tissue, with increased expression in tumors (83). A larger sample of 39 primary and 8 unrelated metastatic pheochromocytoma has shown similar results, with higher levels of p-Akt(Ser473) in primary tumors than normal tissue, and higher levels in metastatic than primary tumors, but these differences were not statistically significant (93). Reports of PTEN immunostaining are less consistent, with reports of both increased (83) and decreased (93) levels in these tumors. Further evidence of altered PI3K/Akt/mTOR signaling is suggested by increased expression of S6K1 and reduced expression of p27^KIP1^ in human pheochromocytoma compared with normal tissue and elevated pS6 in metastatic tumors (93). Evidence also suggests that mTOR signaling may be driven by loss-of-function *TMEM127* (transmembrane protein 127) mutations in some cases: germline *TMEM127* mutations have been identified in a proportion of familial phaeochromocytomas, and in around 2% of apparently sporadic disease, with loss of heterozygosity in tumor specimens (143, 144). Functional TMEM127 appears to be act as a negative regulator of mTOR, with no apparent effects on Akt activation, therefore *TMEM127* mutation may provide an alternative Akt-independent pathway for mTOR overactivation in these tumors (143).

Despite the apparent importance of PI3K/Akt/mTOR signaling in phaeochromocytoma, there has been limited experience and success in targeting this pathway clinically. Use of everolimus inhibitors in four patients with malignant pheochromocytoma showed disappointing results (135). However, dual inhibitors of mTORC1 and mTORC2 show promise in preclinical work. Dual mTORC1/2 inhibitors, unlike rapamycin, are able to significantly decrease Akt Ser473 phosphorylation. mTOR inhibitor AZD8055 significantly reduced the tumor and metastatic burden in athymic nude mice injected with cells of the metastatic mouse pheochromocytoma-derived cell line, MTT, and decreased survival of primary human pheochromocytoma cells in culture (145). Compared to rapamycin, dual mTORC1/2 inhibitor PP242 more effectively increased apoptosis of rat PC12 phaeochromocytoma xenografts, with a more marked increase in levels of pro-apoptotic Bax and reduction in levels of anti-apoptotic Bcl-2, leading to smaller tumors than control mice or those treated with rapamycin (124). Furthermore, dual mTORC1/2 inhibition more effectively suppressed VEGF (vascular endothelial growth factor) and angiogenesis (124). Knockdown of VEGFR-2 in PC12 cells reduced the ability of tyrosine kinase inhibitor sunitinib (which has strong affinity for VEGFRs) to inhibit Akt and mTOR phosphorylation and attenuated sunitinib-induced apoptosis (146). Together these observations suggest a positive feedback loop involving VEGF signaling through VEGF-R2 and PI3K/Akt/mTOR pathway activation. Inhibition of multiple hallmarks of cancer by dual mTORC1/2 inhibitors provides rationale for potential use in human pheochromocytoma.

## Adrenal Medulla: Neuroblastoma

Neuroblastoma is derived from the neural crest and is the most common extracranial cancer of childhood, accounting for approximately 13% of pediatric cancer deaths (147). These tumors most commonly occur in the adrenal medulla, but may occur at any site of the sympathetic nervous system. Neuroblastomas show marked heterogeneity in their clinical course: while some spontaneously regress, others are widely metastatic, resistant to chemotherapy, and carry a high mortality. *MYCN* amplification occurs in 25% of primary tumors, and is an important marker of poor prognosis (148). Mass screening for neuroblastoma once appeared promising, however, such programs are now believed to result in overdiagnosis and overtreatment of biologically favorable tumors, at least a proportion of which would have spontaneously regressed or matured (148, 149).

In recent years, there has been increasing focus on the PI3K/Akt signaling pathway in neuroblastoma. *In vitro* work demonstrates that PI3K/Akt promotes neuroblastoma cell survival, protecting cells from various apoptotic stimuli including cytotoxic chemotherapy (88, 91, 150–152). Akt2 activity may also promote metastatic potential, as silencing Akt2 reduces anchorage-independent growth, migration, and invasion of neuroblastoma cells *in vitro*, and decreases their metastatic potential in mice (153). Furthermore, *in vitro* evidence suggests that Akt signaling interacts with key pathways in neuroblastoma. In neuroblastoma cells, Akt activation indirectly stabilizes N-Myc via negative regulation of GSK3β (154). Furthermore, N-Myc has been reported to regulate a microRNA, miR-184, that in turn targets the 3′UTR of *AKT2* mRNA (155), suggesting that N-myc and PI3K/Akt signaling may form a positive feedback loop. Evidence also suggests that PI3K/Akt is activated downstream of Gastrin-Releasing Peptide (GRP)/Gastrin-Releasing Peptide Receptor (GRPR) and Brain-derived neurotrophic factor (BDNF)/TrkB signaling, which are reported to be overexpressed in human neuroblastoma (151, 153, 156).

A number of studies have used immunohistochemistry to assess Akt activation in human neuroblastoma (Table 2). p-Akt(Ser473) immunostaining is reported to be increased in neuroblastoma compared with normal adrenal medulla (89), and studies have consistently reported detection of p-Akt(Ser473) in a large proportion of tumors (88, 89, 91, 157). p-Akt(Thr308) is less well investigated; however, its expression has been reported in at least 60% of human neuroblastoma (88). Interestingly, p-Akt(Ser473) has been reported to be more strongly expressed in less differentiated cells (157). However, a separate study has reported that while PTEN was lower in undifferentiated neuroblastoma, p-Akt(Ser473) levels were comparable in differentiated and undifferentiated tumors (156).

Activation of the PI3K/Akt pathways is suggested by findings that p-Akt(Ser473) is correlated with downstream mediators, such as p-mTOR and cyclin-D1 (157) as well as potential upstream activators of the PI3K/Akt pathway, such as VEGFR and IGF-1R (91). Interestingly, p-Akt expression has also been reported to be higher in neuroblastoma expressing CD133 (91). CD133 has been identified as a cancer stem cell marker in other tumors and its expression is associated with worse prognosis in neuroblastoma (91, 158). The association between p-Akt and CD133 in human neuroblastoma is potentially important: CD133 appears to promote a stem-cell like phenotype and chemoresistance *in vitro*, and both these actions are at least partially mediated by the PI3K/Akt pathway (91, 158).

In a study of 116 neuroblastoma, p-Akt(Ser473) and p-Akt(Thr308) expression correlated with markers of poor prognosis, such as *MYCN* amplification, changes in 1p36, and stage IV disease. Furthermore, p-Akt(Ser473) and p-Akt(Thr308) expression were associated with worse event-free and overall survival in this sample (88). Notably, p-Akt(Ser473) immunostaining showed fewer associations with prognostic markers and showed a weaker association with survival than immunostaining for p-Akt(Thr308) or for both markers (88). While a second large study failed to show any association between p-Akt(Ser473) immunostaining and prognosis (91), expression of genes downstream of PI3K/Akt signaling has been associated with shorter overall survival (159). In addition, expression of p-Akt(Ser473) is significantly lower in tumors identified by mass screening than in other neuroblastomas (90). As discussed above, tumors identified by mass screening are rarely aggressive, have a substantially better prognosis, and may have otherwise have spontaneously regressed or matured (148, 149); it is possible that lower levels of Akt signaling could contribute to the favorable behavior of these tumors.

## Gastroenteropancreatic Neuroendocrine Tumors

Gastroenteropancreatic neuroendocrine tumors (GEP-NETs) are a heterogeneous group of neoplasms. GEP-NETs are derived from neuroendocrine cells of the gastrointestinal tract and pancreatic islets, with intestinal NETs accounting for approximately 50% of cases, and pancreatic NETs accounting for approximately 30% (160). GEP-NETs are rare, but their incidence appears to be increasing (161). Most cases are sporadic; however, GEP-NET may occur in association with familial cancer syndromes such as MEN1, VHL, NF-1, and tuberous sclerosis. These tumors classically have an indolent course, but presentation often occurs at a late stage with metastases present at diagnosis (162). GEP-NETs can be divided into non-functional and functional tumors, with functional tumors resulting in clinical syndromes due to excessive hormone secretion. Small-intestinal NETs (carcinoids) present with carcinoid syndrome in 30% as a result of secretion of bioactive amines, resulting in an array of symptoms including vomiting and diarrhea (160). Approximately 50% of pancreatic neuroendocrine tumors (P-NET) are functional, producing gastrin (gastrinoma), insulin (insulinoma), glucagon (glucagonoma), or vasoactive intestinal peptide (VIPoma), or more rarely, other peptides/hormones (160). Surgery may cure localized GEP-NET disease, however, systemic therapies are currently limited due to the poor response to traditional cytotoxic chemotherapy. In recent years, there has been increasing interest in targeting PI3K/Akt in these cancers.

Work in GEP-NET cell lines demonstrates that the PI3K/Akt pathway is a promising target for therapy. Several studies have demonstrated that inhibiting this pathway *in vitro* (through PI3K inhibition, p-Akt inhibition, or siRNA) impairs cell proliferation and in some cases survival (163–166). Notably, the effects of Akt inhibition appear to be isoform specific: in human P-NET (BON1) cells, cell viability is impaired by knockdown of Akt1 or Akt3, but promoted by knockdown of Akt2 (165). Furthermore, inhibiting the PI3K/Akt pathway has been shown to reduce the expression of neuroendocrine tumor markers, such as ASCL1 and CgA in pancreatic BON1 cells (166) – this is potentially clinically relevant, as targeting the PI3K/Akt pathway may not only limit tumor cell proliferation or survival, but could conceivably control the troublesome symptoms resulting from hormone oversecretion (166). Evidence also suggests that the PI3K/Akt pathway may promote metastasis and invasion in these tumor cells: knockdown of Akt1, Akt2, or Akt3 impairs neuroendocrine cell invasion (165), and knockdown of PTEN promotes liver metastasis in mouse xenograft models (167).

Gene expression profiling of P-NET has demonstrated that *PTEN* and *TSC2* are downregulated in 50 and 35% of patients, respectively, and downregulation is predictive of poor survival (168). Loss of heterozygosity at the *PTEN* locus is common in P-NET, occurring in 53% of malignant tumors (169). However, *PTEN* mutations are relatively uncommon in these tumors: in an analysis of 68 P-NETs, 7% were found to have mutations in *PTEN* (8% had mutations in *TSC2*, and 1.4% had mutations in *PIK3CA*) (170). The PI3K/Akt pathway is perhaps less well studied in intestinal NET than P-NET, however, in a cohort of 48 well-differentiated small intestinal NET (SI-NET), approximately one-third were found to have genetic alterations in the PI3K/Akt/mTOR pathway, with amplification of Akt1/2 being the most common anomaly in this pathway (171).

Immunohistochemistry is also suggestive of dysregulation of the PI3K/Akt pathway in these cancers. PTEN expression has been reported to be either reduced (168) or show altered subcellular localization (169) in P-NET. Lack of detection of PTEN and Akt2 positivity were both associated with failure to respond to systemic therapy in a mixed group of GEP-NETs, but despite this, Akt2 positivity was associated with longer overall survival (172). Low PTEN was associated with shorter time to progression and disease-free survival in a sample of 137 P-NET (168), and low PTEN expression was independently associated with shorter overall survival and metastasis-free survival in a sample of 160 low and intermediate grade P-NET (173).

Assessment of p-Akt expression in GEP-NET suggests that the PI3K/Akt pathway is active in these tumors (Table 2). In a sample of 46 GEP-NET, immunohistochemistry demonstrated that 61% of tumors were positive for p-Akt(Ser473) (95), and in a mixed sample of NET from various sites, 76% showed positive immunostaining (94). Furthermore, in mixed samples of NET, there was significant association between p-Akt(Ser473) and p-EGFR (94) and between mTOR, PIK3CA, or p-EIF4EBP1 and elevated Ki67 (98). Restriction of the analysis to intestinal NET revealed an association of these proteins with disease-free survival (98). Likewise, western blotting suggests that p-Akt(Ser473) correlates with cyclin D1 expression in P-NET (174), suggesting that these signaling pathways are actively promoting cell proliferation in these cancers, although cyclin D1 did not relate to tumor aggressiveness. Immunohistochemical studies have not demonstrated any significant association between p-Akt(Ser473) immunostaining and prognosis, tumor grade or other clinicopathological variables (94, 95, 97, 98), although this could perhaps reflect high levels of heterogeneity in samples examined. In a small sample of 14 gastrointestinal NET, high p-Akt levels measured by western blotting and a high p-Akt:PTEN ratio showed a non-significant association with shorter overall survival (163). The same study also reports a significant association between p-Akt expression and high levels of serum chromogranin A (CgA) expression (a serum marker of NET), and non-significant associations with the presence of carcinoid syndrome (163). While p-Akt(Ser473) has not convincingly been shown to be associated with prognosis, expression of its downstream target mTOR has been associated with tumor grade or worse prognosis (98, 175), but this has not been found in all studies (176).

There is increasing interest in targeting the PI3K/Akt/mTOR pathway, particularly in P-NET, which has been reviewed by Wolin (177). mTOR inhibitors have been investigated most extensively and have shown promise: a phase III trial of everolimus in advanced, low-, or intermediate-grade P-NET showed prolonged progression-free survival versus placebo (11 months versus 4.6 months) (178). Everolimus has now received Federal Drug Administration (FDA) approval for the treatment of advanced progressive P-NET. Markers of PI3K/Akt activation may be of use as biomarkers for sensitivity to mTOR inhibition in these tumors: cell lines with mutations in *PIK3CA* or *PTEN* are significantly more likely to be sensitive to rapamycin *in vitro*, and cells sensitive to rapamycin show higher baseline levels of p-Akt(Ser473) and p-Akt(Thr308), and greater increases in p-Akt(Ser473) and p-Akt(Thr308) following rapamycin treatment (97). Furthermore, in patients with NET treated on a phase II trial of everolimus and octreotide, pre-treatment and on-treatment p-Akt(Thr308) levels measured by RPPA from fine needle aspirates (FNA) were associated with longer progression-free survival, and increases in Akt phosphorylation from paired samples was associated with response to treatment (97). Interestingly, however there was no association between immunostaining for p-Akt(Ser473) from archival tumor blocks and prognosis (97). There are limited data from clinical research relating to novel inhibitors of the PI3K/Akt/mTOR pathway, although preclinical studies suggest that they may be of interest (177). A phase I trial of an allosteric oral Akt inhibitor, MK-2206, showed that treatment was safe with an acceptable side effect profile, furthermore, p-Akt(Ser473) immunostaining was reduced in post-treatment tumor biopsies compared with pre-treatment biopsies, and partial responses were noted in two patients with P-NET (179).

## Discussion

Overall, a significant weight of evidence suggests that the PI3K/Akt pathway may have critical roles in multiple tumors of endocrine tissues. It is well established that Akt activation is a key mediator of cell proliferation, survival and motility, and, as discussed above, a significant body of *in vitro* work demonstrates that this is also applicable to endocrine cell lines. Furthermore, evidence from animal models, human cancer syndromes and immunohistochemical staining patterns in human endocrine tumors suggest that PI3K/Akt signaling may contribute to tumor progression, and, may even play a role in tumor initiation in some situations. The central role of the PI3K/Akt pathway in regulating many of the hallmarks of cancers makes it attractive as a potential indicator of prognosis, and a potential biomarker for response to therapies – especially in relation to novel agents targeting the PI3K/Akt pathway or downstream targets.

While immunohistochemical studies have undoubtedly provided invaluable insights into PI3K/Akt signaling in human cancers, concerns have been raised about the validity of p-Akt immunohistochemistry for assessment of Akt signaling because phospho-proteins are relatively unstable in tissue samples, with a reported half-life of 20 min (180). Since many studies rely on archival paraffin embedded material and the tumor collection process is not overseen by the researchers, the level of p-Akt activity *in vivo* could be underestimated. Sajjad et al. (110) caution against using cadaveric material for this reason, favoring histologically normal tissue adjacent to tumors as control samples. Furthermore, negative feedback regulation of the PI3K/Akt pathway may lead to strong mTOR staining accompanied by relatively modest p-Akt staining (61). While individual downstream targets are useful as a readout for p-Akt activity *in vitro* where the PI3K–Akt is modulated by specific treatments, use of downstream targets to validate the activation of the PI3K–Akt pathway *in vivo* requires a set of diverse targets, since each target is subject to additional regulation. For example, ERK, S6K, GSK3β, and AMP-activated protein kinase can all promote mTORC1 activity by phosphorylating TSC2 (1). It will also be valuable to identify which downstream targets are of relevance to specific cancers and tumor characteristics.

The vast majority of immunohistochemical studies of endocrine cancers have utilized p-Akt(Ser473) as a marker for Akt activation, with Cell Signaling Technology antibodies being favored. Unfortunately, relatively few papers report which specific p-Akt(Ser473) antibody is used. Currently two of four rabbit monoclonal antibodies (D9E and 736E11) are recommended by the company for immunohistochemistry; there is also a rabbit polyclonal, which has commonly been used for immunohistochemistry. In addition, while it is clear that Akt activation in tumors can be revealed by enhanced p-Akt(Ser473) immunohistochemical staining and outcomes have been supported by western blotting results, exploration of the relative usefulness of p-Akt(Ser473) versus p-Akt(Thr308) as an indicator of Akt activation may be informative. Interestingly, when protein lysates from individuals with *PIK3CA* or *AKT1* mutations were compared with controls by western blotting using p-Akt(Ser473) and p-Akt(Thr308) antibodies, affected individuals showed significantly higher p-Akt(Thr308), but not p-Akt(Ser473) (29). In a study of non-small cell lung cancer, it was found that p-Akt(Thr308), but not p-Akt(Ser43) correlated with phosphorylation of three downstream substrates (PRAS40, TSC2, and TBC1D4), leading the authors to suggest that p-Akt(Thr308) represents a better measure of Akt activity in these tumors (181). However, an immunohistochemical study of colorectal carcinomas found that p-Akt(Ser473) staining correlated with phosphorylation of its downstream substrates GSK-3β and Bad, while p-Akt(Thr308) expression only correlated with phosphorylation of GSK-3β, and not that of Bad (182), perhaps suggesting substrate specific targeting. While in Figure 1, we depict activity of Akt in the absence of serine 473 phosphorylation toward TSC2 and GSK3β targets, Bhaskar and Hay (17) suggested that this may be conformation dependent, and put forward a suggestion that p-Akt(Thr308) may only be active in the membrane-bound form. No study has directly compared p-Akt(Ser473) and p-Akt(Thr308) in cancers of endocrine tissues. The Cancer Genome Atlas Research Network (30) compiled an RPPA data set including p-Akt(Thr308) and p-Akt(Ser473) to show that Akt is activated in *RAS*-like papillary thyroid tumors, however from these data, depicted as a heat map, it can be seen that Akt is not always highly phosphorylated on both sites.

Akt is subject to multiple additional post-translational modifications including phosphorylation at other residues, ubiquitination (lysine 63 linked), sumoylation, and glycosylation in the form of β-d-*N*-acetylglucosamine monosaccharide attachment; Akt2 can additionally be oxidated leading to intramolecular disulfide bonds between cysteine residues (183). These may regulate stability, trafficking, subcellular localization or substrate specificity of Akt. Sumoylation at Lys(276) is essential for Akt activation, which phosphorylates and promotes the activity of the sumoylation machinery, providing a positive feedback loop, which may be damped down by the sumoylation of PTEN (184). Glycosylation may occur at several sites, and Thr(305) and Thr(312) glycosylation may inhibit phosphorylation of Akt on Thr(308) (183). In murine pancreatic β-cells, glucosamine treatment led to decreased p-Akt(Ser473) in favor of *O*-linked *N*-acetylglucosamine at the same residue (185). Therefore, the complexities of regulation of Akt function by post-translational modification mean that we should be cautious in interpreting p-Akt(Ser473) as being fully active Akt.

Combination of PI3K inhibitors and mTOR inhibitors, dual mTORC1/2 inhibition, and mTOR and receptor tyrosine kinase inhibitors are strategies being explored for cancers of endocrine tissues in which the PI3K/Akt/mTOR pathway is hyperactivated (21). In thyroid carcinomas, inhibition of PI3K or mTOR enhances iodine uptake, raising the possibility of enhanced sensitivity to ^131^I therapy in cancers in which this pathway is activated (186). In addition, two types of Akt inhibitors are in clinical development, catalytic ATP-competitive inhibitors that target active Akt, preventing access of phosphatases, and allosteric inhibitors that prevent recruitment to the cell membrane and hence prevent activation. Using such inhibitors, the first type would lead to elevated levels of phosphorylated Akt, while the second type would prevent phosphorylation. Difficulties arise with activation of feedback pathways and compensatory pathways subsequent to Akt inhibition. Such issues, along with off target effects, need to be considered to ascertain dosing schedules that are sufficient to give long term inhibition. Further, it will be important to ascertain what level of active Akt would be required in a particular tumor before targeting of the PI3K/Akt pathway is likely to be of benefit (187).

Rather than selecting patients for treatment at random, better outcomes may result from a personalized approach to therapy by utilizing predictive biomarker screening strategies. To address this, Yan et al. (188) determined a set of biomarkers that can be used to predict patients likely to benefit from targeted Akt inhibition using GDC-0068, an inhibitor of the ATP competitive type. Of 100 protein and phospho-specific antibodies used in RPPA of lysates from tumor cell lines (PC3, prostate; U87, glioblastoma; MCF7.1, breast), p-Akt(Ser473) and p-Akt(Thr308) were elevated after treatment as expected, whereas substrates downstream of Akt, PRAS40, GSK-3β, mTOR, eIF4G, ATP citrate lyase, FoxO3A and S6, showed decreased phosphorylation. However, the array also revealed feedback activation of the MAPK pathway, with elevated p-ERK, and enhanced levels of HER3. Similar biomarker responses were obtained in mouse xenograft models and in human tumor material from a “first in human” trial of GDC-0068 in patients with advanced solid tumors. Interestingly, the tissue was removed by core needle biopsy and stroma and tumor cells were laser captured separately for RPPA analysis and validated by immunohistochemistry for p-Akt(Ser473), p-Akt(Thr308) and p-PRAS40(Thr246). While p-Akt(Ser473) and p-Akt(Thr308) were elevated in both tumor and stroma, p-PRAS40(Thr246) was only reduced in the tumor tissue, illustrating the importance of investigating the tumor cell population specifically. The authors propose that use of a biomarker set gives more robust readout for Akt inhibition than use of a single biomarker. Moreover, FNA or core needle biopsies allow comparison of pre-treatment and on-treatment functional proteomics by quantitative RPPA for components of the PI3K/Akt/mTOR pathway, particularly if tissue collection is optimized to maintain antigenicity of phosphorylated proteins such as p-Akt by fixation in cold formalin within minutes of removal from the patient, which is more successful for small core biopsies than larger tissue specimens (189, 190). Sampling replicates can be used to account for tumor heterogeneity, but microdissection may be needed for tumors with stromal or other tissue elements. With careful consideration of these limitations, these methods may be developed further to allow prediction of drug response.

A full understanding of how Akt and its targets are regulated is important for the development of effective targeted therapies, rational development of therapeutic combinations, and a better identification of biomarker panels in tumors of endocrine tissues. Future work will need to explore the efficacy of further treatment combinations, such as combinations with XIAP or ERK inhibition, and it will be important to explore how best to promote apoptotic responses over cytostatic effects of treatment without engendering intolerable toxicity. Alongside these developments, biomarkers of the PI3K/Akt pathway have the potential to predict and monitor tumor response. Overall, current knowledge suggests that this pathway plays a substantial role in tumors in endocrine tissues, and hence lends strong justification for further work in this area.

## Author Contributions

HR and AH contributed equally to the conception and design, literature review, and final written review article.

## Conflict of Interest Statement

The authors declare that the research was conducted in the absence of any commercial or financial relationships that could be construed as a potential conflict of interest.
